# Associations between COVID-19 Pandemic-Related Overtime, Perceived Chronic Stress and Burnout Symptoms in German General Practitioners and Practice Personnel—A Prospective Study

**DOI:** 10.3390/healthcare12040479

**Published:** 2024-02-16

**Authors:** Lucas Küppers, Julian Göbel, Benjamin Aretz, Monika A. Rieger, Birgitta Weltermann

**Affiliations:** 1 Institute of General Practice and Family Medicine, University Hospital Bonn, University of Bonn, Venusberg-Campus 1, 53127 Bonn, Germanybirgitta.weltermann@ukbonn.de (B.W.); 2 Institute of Occupational and Social Medicine and Health Services Research, University Hospital Tuebingen, 72074 Tuebingen, Germany; monika.rieger@med.uni-tuebingen.de

**Keywords:** COVID-19, burnout, chronic stress, overtime, general practitioners, practice personnel

## Abstract

Background: The mental burdens of general practitioners (GPs) and practice assistants (PrAs) during the COVID-19 pandemic are well investigated. Work-related conditions like overtime are known to contribute to perceived chronic stress and burnout symptoms. However, there is limited evidence regarding the specific mechanisms, which link pandemic-related overtime, chronic stress and burnout symptoms. This study used data from the IMPROVE*job* trial to improve psychological well-being in general practice personnel. Methods: This prospective study with 226 German GPs and PrAs used the baseline (pre-pandemic: October 2019 to March 2020) and follow-up data (pandemic: October 2020 to April 2021) of the IMPROVE*job* trial. Overtime was self-reported as hours above the regular work time. Perceived chronic stress was assessed using the Trier Inventory for the Assessment of Chronic Stress Screening Scale (TICS-SSCS), while burnout symptoms were evaluated using a short version of the Maslach Burnout Inventory (MBI). A mediation analysis investigated the differences of the three main variables between pre-pandemic and pandemic periods. Results: Burnout symptoms increased significantly from baseline to follow-up (*p* = 0.003). Overtime correlated positively with burnout symptoms (Total Effect: 0.13; 95% CI: 0.03, 0.23). Decomposition of the total effect revealed a significant indirect effect over perceived chronic stress (0.11; 95% CI: 0.06, 0.18) and no significant direct effect (0.02; 95% CI: −0.08, 0.12), indicating a full mediation. Conclusion: In this large longitudinal study, pandemic-related overtime led to significantly higher levels of burnout symptoms, linked by a pathway through perceived chronic stress. Future prevention strategies need to aim at reducing the likelihood of overtime to ensure the mental well-being of practice personnel.

## 1. Introduction

### 1.1. Chronic Stress in General Practice

Chronic stress is a global public health burden and is prevalent across the general population [1]. Data for the German population in a sample of 5850 participants showed a chronic stress prevalence of 13.9% for women and 8.2% for men [2]. Chronic stress itself is potentially leading to mental disorders [3] and/or organic diseases such as cardiovascular diseases [4]. A systematic review of 41 studies revealed that workplace-related factors such as work intensity are some of the most frequent causes of stress in the general population [5].

Also, chronic stress is very common among healthcare professionals, especially among general practitioners and general practice personnel who face multiple challenges in their daily work [6]. This has been shown in many countries worldwide. A recent cross-sectional study with 9526 general practitioners (GPs) across ten European and former Commonwealth countries indicated that 68% of German GPs perceive their work as very or extremely stressful. The prevalence varied markedly between countries with British (71%) and German GPs (68%) indicating the highest levels of stress while less than 50% of Swiss and Dutch GPs rated their job as stressful [7]. These differences are suggesting that the healthcare systems with their individual organizational structure and financing as well as healthcare reforms and regulations might play a role [8,9]. Regarding German general practice personnel, Viehmann et al. revealed in a sample of 136 German general practices that practice personnel is up to twice as prone to high chronic stress compared to the average population. The highest stress levels were observed especially among female physicians followed by practice assistants with less work experience [6]. High levels of chronic stress among physicians are associated with more hours of working [6], unhealthy practice culture [10], and challenges of practice management [11].

### 1.2. Burnout in General Practice

While chronic stress is important, it also needs to be considered as part of a spectrum of indicators of psychological well-being. For example, it is widely recognized that chronic stress and burnout symptoms are closely linked [12]. The World Health Organization defines burnout as a combination of exhaustion, feelings of negativism related to one’s job, and reduced professional efficacy [13]. A systematic review of 182 studies including 109,628 physicians from all specialties in 45 countries found a remarkable prevalence of overall burnout symptoms of up to 80.5% [14]. According to another systematic review of 60 studies across 29 countries, the prevalence of burnout among GPs ranged from 6% to 33% with GPs from China, Denmark and the UK showing the highest levels of burnout symptoms in the comparison by country [15]. As for chronic stress, different burnout levels per country are often associated with the local healthcare systems [16]. Practice assistants (PrAs) and nurses in primary care settings also reported high levels of emotional exhaustion, low personal accomplishment, and chronic stress [17,18]. According to Bozorgmehr et al., workload and time pressure are some of the main predictors of stress in PrAs [19].

### 1.3. Sources for Chronic Stress and Burnout

**Figure 1:**
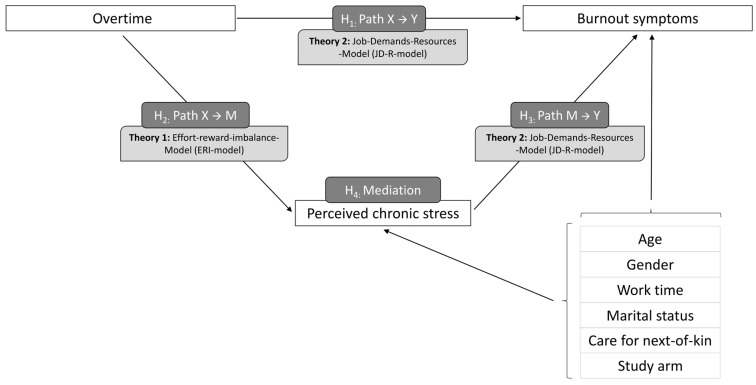
Research model of this study.

Excessive work demands are some of the main triggers of chronic stress and burnout according to the Effort-Reward Imbalance (ERI) model (Theory 1, Figure 1) and the job demand resources model (Theory 2, Figure 1), which is one of the most common models explaining burnout in the occupational context [20,21]. This is especially applicable in highly burdened occupational groups such as primary care physicians and their personnel [6,17,22]. These quantitative and emotional demands [23] are triggered, among others, by confrontation with other people (e.g., patients), negative feelings at work (emotional demands), and incongruity between the number of tasks and the time available to perform them, often leading to overtime (quantitative demands) [24]. With regard to chronic stress, the ERI model is a theoretical framework aiming at comprehending the impact of workplace stress (e.g., overtime) on health. The model proposes that health issues, especially mental health disorders, may arise from stress due to an inequity between the effort employees exert in their work and the rewards they receive. Particularly during challenging times in practices, GPs tend to work more hours in practice without getting adequate rewards (e.g., remuneration) [21].

Work-related conditions predict effort and reward of GPs and also contribute to their mental well-being according to a recent meta-analysis [25]. This is grounded in the specifics of the GP workplaces, as GPs have to juggle not only multiple responsibilities in patient care but also various administrative tasks and practice management while dealing with time constraints and limited resources. A cross-sectional study with 226 German GPs found that keeping medical records up-to-date was a main source of chronic stress for participants [26]. This is in line with findings from Gardner et al., who showed that the extensive use of medical information systems can lead to stress and burnout especially in primary care-oriented physicians [27]. Besides overnight calls, high work intensity, and work-privacy conflicts, overtime is suggested to be one of the most prominent work-related sources of both chronic stress and burnout [6,28]. According to a study among 7288 US physicians and 3442 working US adults, 37.9% of physicians and 10.6% of the control population worked more than 60 h per week. Those physicians who worked more than 60 h per week had a significantly higher risk of developing burnout symptoms [28].

### 1.4. Mental Health Impact of the COVID-19 Pandemic

In addition to risk factors at the individual and practice level, also hardships at the global level (= exogenous shocks) are relevant for the mental health of general practice personnel [29]. For example, the recent COVID-19 pandemic increased the workloads of frontline healthcare professionals and their risk for various mental disorders [30,31]. A cross-sectional study by Moro et al. among Italian healthcare workers from all branches revealed, that 37.9% of the participants were suffering from psychological stress during the first phase of the pandemic [32]. In November 2020, 94% of the COVID-19 patients in Germany were treated in primary care [33]. Handling the bulk of COVID-19 outpatients, GPs suffered from a high psychological burden and challenging working conditions [34,35,36]. A systematic review by Jefferson et al. including 31 studies from multiple countries worldwide stated the tremendous decrease in GP well-being during the pandemic with significant increases in stress, burnout, depression, and anxiety disorders [29].

### 1.5. The IMPROVEjob Trial

Various intervention studies aimed at improving GPs’ work situations focussing either on the individual or the work conditions [37]. Individual prevention like stress management and reduction as well as improvement in self-care have already been addressed [38]. However, organizational work structures should be changed first before targeting the individual level [39]. A systematic review with 39 studies by Montano et al. investigated interventions aimed at improving organizational workplace conditions and showed that comprehensive interventions including multiple aspects like organizational and worktime-related factors at the same time showed the highest success rates [40]. A recent narrative review with 108 randomized controlled intervention trials included 56 RCTs covering burnout and 14 RCTs covering psychosocial work stressors in healthcare workers concluded that there are frequent barriers to participate in workplace interventions (e.g., time pressure, no support by employers) and that most interventions only provided short-term benefits on mental health of healthcare workers [41].

Given the high prevalence of chronic stress in German practice personnel, the cluster-randomized controlled IMPROVE*job* trial aimed at improving practice work situations by optimizing leadership, practice processes, and communication within the practice team and with patients. The main outcomes were to improve job satisfaction and reduce perceived chronic stress in the surveyed general practice personnel. Applying live training sessions, an online toolbox and trained facilitators, the IMPROVE*job* intervention used modern learning techniques to achieve structural and behavioral changes in the study population. The study was conducted by an interdisciplinary research consortium comprising clinician scientist experts from family medicine and experts from occupational medicine, psychosomatic medicine, operations research, epidemiology, and health promotion. The study protocol, baseline and follow-up data of the intervention are published elsewhere [17,42,43]. The baseline data showed high job satisfaction and high levels of perceived chronic stress among general practice personnel [17]. The results of the intervention showed no effects on job satisfaction and perceived chronic stress at follow-up, but the results were biased by the COVID-19 pandemic [43].

### 1.6. Theory, Hypothesis, and Research Question

The data for this prospective study was derived from the IMPROVE*job* trial and analyzed the role of perceived chronic stress as a possible mediator between overtime and burnout symptoms among German general practice personnel in the context of the COVID-19 pandemic. Based on the existing theory and previous research (see Figure 1 for our research model), we hypothesize that there is a correlation between overtime and burnout symptoms (H1), overtime and perceived chronic stress (H2), and perceived chronic stress and burnout symptoms (H3). In addition, we hypothesize that perceived chronic stress might have a mediating role in the development of burnout symptoms due to overtime (H4). Accordingly, we formulated the research question: ‘What role does perceived chronic stress play in the causal chain between overtime and burnout symptoms?’

## 2. Materials and Methods

### 2.1. Study Design and Setting

**Figure 2:**
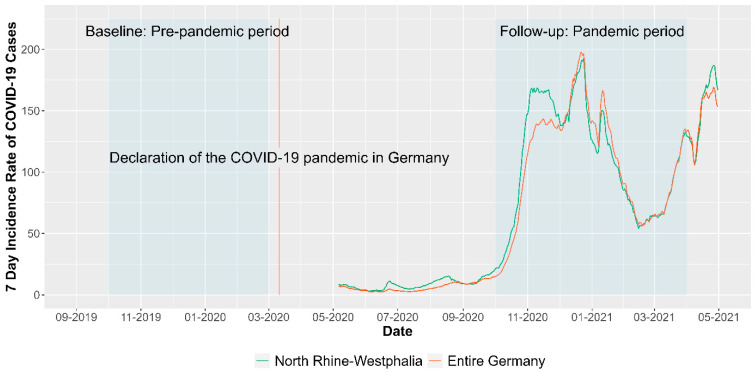
Seven-Day Incidence Rate of COVID-19 Cases during data collection period in Germany and the study region North Rhine-Westphalia. Note: Data for illustration was derived from Robert Koch Institute 7-day incidences according to federal states (21 April 2023) [44].

This prospective study used the baseline (pre-pandemic period: October 2019 to March 2020) and follow-up (pandemic period: October 2020 to April 2021) data of the IMPROVE*job* trial conducted in the federal state of North Rhine-Westphalia, Germany. The timeline is outlined in Figure 2. The cluster-randomized controlled trial (Registration number: DRKS00012677) investigated the effectiveness of the multimodal participatory IMPROVE*job* intervention on improving job satisfaction and reducing chronic stress of general practice personnel including both GPs and PrAs [17,42].

Addressing an important issue in general practice personnel, the trial was funded by the German Federal Ministry for Education and Research. Secondary outcomes included, among others, overtime, burnout symptoms, and perceived chronic stress. These outcomes have not been analyzed in the follow-up paper within the context of the COVID-19 pandemic [43]. The data included the intervention and control groups. By using the baseline (pre-pandemic period) and follow-up data (pandemic period), this study considered the COVID-19 pandemic as a natural intervention, encompassing the first year of the pandemic (March 2020 to April 2021). A mediation analysis investigated if the pathway between overtime and burnout symptoms is mediated by perceived chronic stress.

The IMPROVE*job* trial was approved by the Ethics Committee of the Medical Faculty of the University of Bonn (reference number 057/19, date of approval: 20 February 2019). In addition, the Ethics Committees of the Medical Association North-Rhine (Lfd-Nr.: 2019107) and of the Medical Faculty, University Hospital of Tuebingen (Project-No.: 446/2019BO2) also voted positively. Each participating practice team member provided written informed consent.

### 2.2. Recruitment and Analysis Population

Practices were eligible for inclusion if their owner was registered as a GP of the Association of Statutory Health Insurance Physicians of North Rhine, with or without affiliation as a teaching practice of the University of Bonn or the University of Cologne. A second inclusion criteria required the informed consent for the study participation by the the practice owner and at least one practice assistant. GPs, that had been involved in the development of the IMPROVE*job* intervention or had participated in the feasibility study of the intervention, were excluded. Practices facing circumstances such as an imminent relocation of the practice or the retirement of the practice owner were also not eligible for inclusion. Urban and rural general practices, single and group practices as well as teaching and non-teaching practices of the North Rhine region in Germany were included in the IMPROVE*job* trial. Randomization was conducted at practice level with practices acting as clusters. The intervention group received the IMPROVE*job* intervention after answering the baseline questionnaire while the control group received the intervention after completion of the study. Data for baseline and follow-up was collected via paper-pencil questionnaires. The questionnaires included questions on socio-demographic characteristics and items to assess general practice personnels’ working hours including overtime, perceived chronic stress levels, burnout symptoms, and further psychosocial factors (see Table 1 for absolute and relative socio-demographic frequencies).

**Figure 3:**
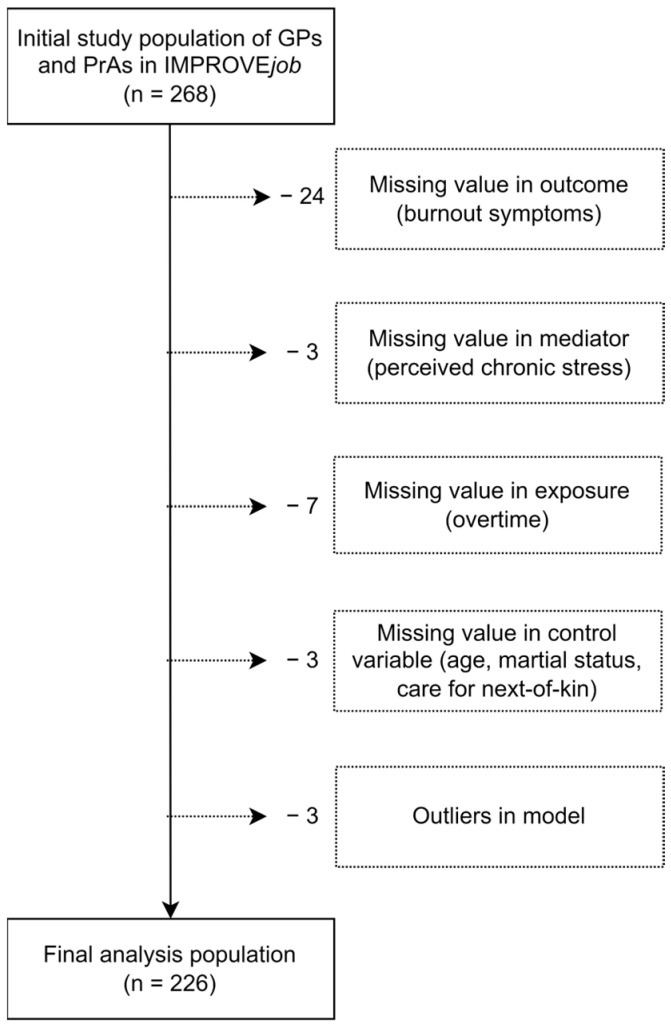
Construction of the analysis population based on the IMPROVE*job* study population of GPs and PrAs in Germany.

For this study, 42 cases from the original study population of IMPROVE*job* (n = 268) had to be excluded due to missing values in the outcome variables or due to outliers in the model (Cook’s D > 3) leading to a final analysis population of 226 participants, of whom 68 were GPs and 158 were PrAs (Figure 3).

### 2.3. Outcome Variable: Pandemic-Related Changes in Burnout Symptoms

Burnout symptoms were assessed by a short version of the Maslach Burnout Inventory (MBI), involving the utilization of two items. These items focus on emotional exhaustion (‘I feel burned out from my work.’) and depersonalization (‘I have become more callous toward people since I took this job.’) [45]. The short version of the MBI has shown its effectiveness and validity in previous studies [46]. The concise measure has demonstrated its effectiveness in gauging the likelihood of experiencing high levels of burnout symptoms among physicians and medical students. Those among the 10,525 participants who answered ‘a few times a week’ on the single item measures for emotional exhaustion or depersonalization had a more than 90% possibility of high burnout degrees in each domain on the regular MBI [47]. The two-item MBI short version was chosen because it covers two important factors for medical professionals: the emotional demands of their work and potential feelings of depersonalization towards their patients. We tested the temporal stability of the used MBI short version, which showed good internal consistency between baseline and follow-up measurements (Cronbach’s alpha = 0.752).

The scoring system ranges on a 7-point Likert scale from 0 (indicating never) to 6 (indicating every day) resulting in a sum score taking emotional exhaustion and depersonalization into account [48]. For the final analysis, burnout symptoms were measured at baseline (pre-pandemic) and follow-up (pandemic) (Table 1). We calculated the difference between the baseline and follow-up burnout symptoms (pandemic-related change denoted with delta). Positive values indicate increases in burnout symptoms, while negative values indicate a decline. The difference in burnout symptoms was included in the mediation analysis as the outcome variable.

### 2.4. Independent Variable: Pandemic-Related Changes in Overtime

Overtime among general practice personnel was surveyed by the following item: ‘How many hours of overtime do you have to work each week due to work process issues?’. Participants received a free text box to enter a number of hours and minutes. Hourly data were included in the final analyses. Values >30 min were rounded up to the next full hour. We calculated the difference between the baseline and follow-up overtime. This change in overtime represented the independent variable in the mediation analysis (Table 1).

### 2.5. Mediator Variable: Pandemic-Related Changes in Perceived Chronic Stress

The German short version of the Trier Inventory for the Assessment of Chronic Stress Screening Scale (TICS-SSCS) evaluated perceived chronic stress over the previous three months. The original instrument is based on the theory that stress is caused by the strain on personal resources in a person’s interaction with their environment. Subsequently, chronic stress develops gradually, typically over a longer time course and without a recognizable beginning. The original TICS comprises six dimensions including work overload, job dissatisfaction, social stress, lack of social recognition, worry/concern, and stressful memories [49].

The German short version TICS-SSCS consists of 12 items (e.g., ‘In the last three months, how often did you experience fear of not being able to perform your duties?’) with responses rated on a 5-point Likert scale and provides a global measure of perceived chronic stress. The individual item scores were combined into a sum-score ranging from 0 to 48, where 0 indicates ‘never stressed’ and 48 represents ‘very often stressed’ [50]. The TICS-SSCS has excellent internal validity, as indicated by a Cronbach’s alpha of 0.91 [49]. The difference between baseline and follow-up perceived chronic stress was calculated and used as the mediator in the mediation analysis (Table 1).

### 2.6. Control Variables

As potential confounding factors age, gender, work time model (full-time/part-time), marital status, and care for next-of-kin (yes/no) were included in the mediation analysis. In addition, a control variable differing between participants of the intervention and control groups was included (Table 2). In prior studies, younger age and female gender were associated with higher levels of chronic stress [6], while working full-time leads to increased work-privacy conflicts [51]. Marriage can protect from mental disorders [52] while caring for next-of-kin is associated with an increased risk for burnout [53].

**Table 1:** Pandemic-related overtime, perceived chronic stress and burnout symptoms: baseline (BL), follow-up (FU) and differences between baseline and follow-up (Δ).

	Total Sample (*n* = 226)	
Variable	Mean (SD)	*t*-Test (*p*-Value)
Overtime (BL)	1.34 (2.16)	
Overtime (FU)	1.42 (2.16)	
Overtime (Δ)	0.08 (2.26)	0.5 (0.62)
TICS-SSCS (BL)	19.11 (8)	
TICS-SSCS (FU)	18 (8.42)	
TICS-SSCS (Δ)	−1.11 (7.04)	−2.36 (0.02)
MBI (BL)	5.56 (1.99)	
MBI (FU)	5.92 (2.02)	
MBI (Δ)	0.36 (1.79)	3.04 (0.003)

Note: TICS-SSCS = Trier Inventory for the Assessment of Chronic Stress; MBI = Short version of the Maslach Burnout Inventory.

### 2.7. Statistical Analysis

To test the association between pandemic-related overtime, perceived chronic stress, and burnout symptoms, we applied a mediation approach using Model 4 of the PROCESS macro by Hayes, which employs ordinary least squares (OLS) regression. This method produced unstandardized path coefficients to assess total, direct, and indirect effects. To calculate confidence intervals and inferential statistics, bootstrapping with 5000 samples, along with heteroscedasticity-consistent standard errors was utilized. Effects were considered significant if the confidence interval did not encompass zero. [54,55] The mediation model applies two separate regression equations (OLS) with the independent variable (*X*), the mediator variable (*M*) and the outcome variable (*Y*):

(1) $$M=i_{1}+aX+\varepsilon_{M}$$

(2) $$Y=i_{2}+\gamma 'X+\beta M+\varepsilon_{Y}$$

This approach includes each of the three paths of interest simultaneously in one model, namely the path between overtime and burnout symptoms (*X* → *Y*), the path between overtime and perceived chronic stress (*X* → *M*), and the path between perceived chronic stress and burnout symptoms (*M* → *Y*) (Figure 1).

The total effect can be decomposed into the indirect effect (=mediating effect over perceived chronic stress) and the direct effect (=effect of overtime on burnout symptoms under the consideration of the mediating effect). The model was calculated for the total analysis population controlled for all confounding variables simultaneously. In a sensitivity analysis, the model was additionally estimated for GPs and PrAs separately. To assess multicollinearity, we evaluated the variance inflation factor (VIF), which demonstrated that it did not bias our estimations (VIF < 5).

Statistical analyses were conducted with SPSS Statistics for Windows version 26 (IBM Corp., Armonk, NY, USA). The significance level was set at *p* < 0.05.

## 3. Results

### 3.1. Descriptive Results

The final sample comprised 226 participants, of whom 69.9% were PrAs. The mean age of the participants was 45 years and 86.7% were female. More than half of the population worked full-time. The majority were married or lived in a relationship. Nearly a quarter (21.2%) reported to care for a next-of-kin (Table 2). During the pandemic, the average overtime increased from 1.34 h at baseline to 1.42 h at follow-up (*p* = 0.62). The severity of burnout symptoms increased by 6.47% (*p* = 0.003), while the average levels of perceived chronic stress decreased by 5.81% (*p* = 0.02) (Table 1).

### 3.2. Mediation Analysis of Pandemic-Related Changes in Overtime, Perceived Chronic Stress and Burnout Symptoms

**Figure 4:**
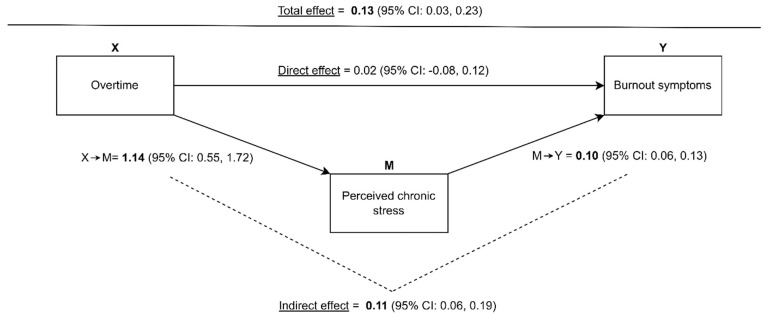
Results of the mediation analysis with decomposition of the total effect into the direct and indirect effects.

The total effect of the mediation analysis showed a significant association between overtime and burnout symptoms (Total Effect: 0.13; 95% CI: 0.03, 0.23, Figure 4). The decomposition into the direct and indirect effects revealed a significant indirect effect (Indirect Effect: 0.11; 95% CI: 0.06, 0.18, Figure 4). The indirect effect (=mediating pathway) is the combined effect of both indirect paths, namely from overtime on perceived chronic stress (*X* → *M*), and from perceived chronic stress on burnout symptoms (*M* → *Y*). However, when considering the indirect effect, no direct effect (*X* → *Y*) was observed between overtime and burnout symptoms (Direct Effect: 0.02; 95% CI: −0.08, 0.12, Figure 4), which the literature defines as a full mediation [56]. Thus, in our analysis model, the association between overtime and burnout symptoms among general practice personnel is completely explained by the pathway through perceived chronic stress. The only significant correlation between a main variable and a covariable was between perceived chronic stress and female gender (*p* = 0.02). The variable differing between participants of the intervention and control groups showed no differences between both sub-populations. The sensitivity analysis confirmed the results from the mediation analysis for both GPs and PrAs (Table 3).

## 4. Discussion

This study investigated associations between overtime, perceived chronic stress, and burnout symptoms among German general practice personnel during COVID-19 using a mediation model. The mediation model confirmed two of our hypotheses by revealing the relationship between overtime and perceived chronic stress (H2) as well as between perceived chronic stress and burnout symptoms (H3). In the study population, pandemic-related overtime and perceived chronic stress increased the likelihood of higher levels of burnout symptoms, confirming our hypothesis (H4). Statistically, the pathway between COVID-19 pandemic-related overtime and the severity of burnout symptoms was fully mediated by perceived chronic stress. According to the definition of a full mediation, the total effect between overtime and burnout was disentangled, leading to a rejection of one of the initial hypothesis (H1). While the mean perceived chronic stress of the population decreased, the sub-population of GPs and PrAs with increased perceived chronic stress is driving the statistical model. The results were consistent for GPs and PrAs, which suggests that overtime and perceived chronic stress play an important role in the development of burnout for both professional groups. Our findings highlight the relevance of overtime and perceived chronic stress in developing burnout symptoms among healthcare professionals and have important implications for strategies to prevent burnout [12].

The results of the indirect paths of the mediation analysis are in line with previous findings. A recent cross-sectional study among 3236 GPs from China found that 58.56% of the participants suffered from high levels of occupational stress with overtime being directly linked to the development of occupational stress [57]. Besides causing chronic stress, overtime also leads to an increased intention of GPs to work part-time according to a quantitative study from the UK: in the time period between 1998 and 2020, the working hours dropped by 25.37% [58]. Another cross-sectional survey with a sample of 320 German GPs identified out-of-hours care as an important stressor in everyday practice and a reason for lower job satisfaction. Almost 80% of the surveyed GPs agreed that less out-of-hours care would improve their overall job satisfaction [59]. This is important in the context, that German GPs are working the most hours per day and are most unsatisfied compared to their other European and US-American colleagues [60]. Overtime increased during the pandemic to an average of 2.01 h in a setting of 3669 German healthcare workers from all specialties [61]. A cross-sectional study among 5502 Italian healthcare workers showed a direct link between pandemic-related overtime and psychological distress [32]. Similarly, stress levels were related to more burnout symptoms in a US study with 579 general internal medicine physicians [62]. In line with the job demand resources model, the COVID-19 pandemic subsequently led to excessive quantitative (e.g., overtime) and emotional demands (e.g., treating more severely ill patients) in GPs with chronic stress and burnout as subsequent adverse mental health outcomes [20,35].

Burnout is a multidimensional construct and is determined by situational and individual factors [45]. Shanafelt et al. reported an increase in burnout symptoms among 2440 US American physicians from 43.9% in 2017 to 62.8% in 2021, which was partly attributed to the chronicity of the COVID-19 pandemic, short staffing, and/or negative economic consequences on the individual or societal level. Alongside emergency medicine practitioners and pediatricians, GPs had the highest risk for burnout symptoms [63]. Similarly, the systematic review by Jefferson et al. found an increase in burnout symptoms up to 46% in GPs worldwide due to the pandemic [29]. The presence of burnout symptoms in GPs is associated with increased sick leave days, enhancing the arising shortage of caregivers in day-to-day health care delivery [16]. A cross-sectional study with 5197 US primary care physicians by Sinsky et al. showed that burnout was directly linked to the intention to leave the job. In detail, 25% of the physicians surveyed voiced the intention to leave their current position in the next 24 months [64]. In addition, a systematic review and meta-analysis of 25 cross-sectional studies by Shen et al. found that lower salaries, low job satisfaction and bad morale caused turnover intentions in 46% of GPs between 1988 and 2019 [65]. A recent meta-analysis of 45 observational studies highlighted the increased burnout levels of physicians worldwide due to the pandemic, with an overall burnout prevalence of 60.7% during the early pandemic phase and 49.3% in later periods. Physicians from the Middle East and North Africa showed the highest burnout levels closely followed by their European colleagues [66]. In the context of this alarmingly high prevalence, overtime has been considered to be one of the main predictors for the development of pandemic-associated burnout symptoms according to a review including five studies from the US and Europe [67]. Pandemic-related overtime can be considered a situational factor, while individual factors such as over-involvement with patients, lack of coping strategies, and higher degrees of neuroticism contribute independently to the development of burnout and—in addition—increase the vulnerability for situational factors [68,69,70]. Despite inconsistent findings in the literature, some studies show that burnout not only affects the mental and physical health of physicians but also influences patient care, potentially leading to medical errors and reduced quality of care [71]. For the United States, it is estimated that around $4.6 billion in financial losses per year are attributable to physician turnover and absence of work due to burnout [72]. This emphasizes the urgency of addressing burnout, not only for the wellbeing of physicians but also for the safety of patients and the effectiveness of healthcare.

The problem of overtime and chronic stress is not easily solved, as a multitude of factors is responsible. Shanafelt et al. emphasize different organizational aspects to counteract burnout and promote well-being in physicians. As a first step, practices and hospitals need to acknowledge the presence of burnout and assess the well-being of physicians regularly. Besides effective leadership, practicing cohesiveness and community at work can significantly contribute to physicians’ health. Employers’ flexibility in allowing physicians to tailor their work hours and allow for better work-life integration is also crucial. Finally, providing physicians with tools to ameliorate self-care and resilience can further help to prevent burnout [73].

Overall, our findings are worrisome considering the already existing and further worsening staff shortage in European and other healthcare systems [74,75,76]. A nationwide longitudinal study in Hungary observed a decrease in GP practices by 7.7% between 2007 and 2016 [74]. These tendencies might ultimately lead to inequities in access to healthcare as well as shortages of medical services [74,77]. While family medicine is still a rewarding career for young residents, as it offers high autonomy in diverse work settings and long-term doctor-patient relationships, the design of workplaces and working conditions needs to respect the described associations to psychological well-being [78,79]. A recent qualitative study with semi-structured interviews of 96 German GPs suggested actions such as upgrading the GP position, creating interdisciplinary outpatient care centers and reforming GP training to secure future primary care [76].

Our study intends to sensitize the healthcare community and health policy makers on the vulnerability of frontline healthcare professionals in a primary care setting during a pandemic. The theoretical implications of this study add to the existing ERI model and the job demand resources model. The results reveal the relationship between occupational stressors and mental health disorders, aggravated by a worldwide health crisis. The practical implications of our research emphasize the need for workshops, specific trainings, optimized practice management, reduced work demands, and less overtime for GPs and general practice personnel, especially in periods of crises. This is in line with the results of a recent review by Shiri et al., suggesting that workplace interventions should be established as mandatory routine programs in the work environments of healthcare workers [41]. By that, optimized work conditions might have the potential to safeguard general practice personnel from adverse health outcomes, especially burnout symptoms.

To the best of our knowledge, this is one of the first studies to assess the effects of COVID-19 pandemic-related overtime on mental health outcomes among German general practice personnel. The results stem from a rather large data set derived in a primary care setting. Nevertheless, the findings should be interpreted in the context of some limitations. The study specifically targeted a particular regional setting in Germany, which necessitates caution when generalizing the results to the German or European-wide context. The decrease in perceived chronic stress at follow-up is likely explained by the heterogeneity of the practice population with some suffering from overtime and consecutive perceived chronic stress, while others had fewer practice duties during the first phase of the pandemic. Besides overtime, other occupational factors may influence the pathway, however, such data were not available in the IMPROVE*job* data set. Future work in representative GP and practice populations should aim for longitudinal data and respect for the heterogeneity of practice work settings.

## Figures and Tables

**Table 2:** Sociodemographic characteristics of the general practice personnel.

	Total Sample (n = 226)
Variable	
Female, n (%)	196 (86.7)
Age in years, mean (SD)	45 (12)
General practice assistants, n (%)	158 (69.9)
Working full-time, n (%)	123 (54.4)
Living in a relationship/married, n (%)	183 (81.0)
Care for next-of-kin, n (%)	48 (21.2)
Control group, n (%)	119 (52.7)

**Table 3:** Results of the mediation analysis (total sample) and the sensitivity analyses (subpopulations) with decomposition of the total effect into the direct and indirect effects.

	Total Sample (*n* = 226)		GPs (*n* = 68)		PrAs (*n* = 158)	
	Coefficient	95% CI	Coefficient	95% CI	Coefficient	95% CI
Total Effect	0.13	0.03, 0.23	0.05	−0.16, 0.26	0.34	0.12, 0.57
Direct Effect	0.02	−0.08, 0.12	−0.03	−0.29, 0.23	0.17	−0.05, 0.38
Indirect Effect	0.11	0.06, 0.19	0.08	0.01, 0.17	0.17	0.08, 0.29

## Data Availability

The original contributions presented in the study are included in the article, further inquiries can be directed to the corresponding author.
